# Epigenetics in B‐CLL

**DOI:** 10.1155/ijog/5877313

**Published:** 2026-02-18

**Authors:** Alexandra Chu, Flavia Soto, Rodrigo Hurtado, Carlos A. Tirado

**Affiliations:** ^1^ The International Circle of Genetic Studies Project New York Chapter, New York City, New York, USA; ^2^ The International Circle of Genetic Studies Project Chiclayo Chapter, Chiclayo, Peru; ^3^ Department of Pathology, Renaissance School of Medicine at Stony Brook University, Stony Brook, New York, USA

## Abstract

B‐cell chronic lymphocytic leukemia (B‐CLL) is the most common hematological malignancy in adults. Its clinical course is heterogeneous, ranging from indolent forms with slow progression to aggressive variants refractory to conventional treatment. In recent years, it has been shown that epigenetic alterations, such as DNA methylation, histone modifications, and regulation by noncoding RNAs, especially microRNAs (miRNAs), play a central role in the prognosis of this disease. For this reason, the analysis of epigenetic mechanisms has become an essential approach both to understand the progression of B‐CLL and to predict therapeutic response and patient survival.

## 1. Introduction

B‐cell chronic lymphocytic leukemia (B‐CLL) is a lymphoid malignancy marked by the abnormal growth and proliferation of mature CD5 positive B cells in the blood, lymphoid tissues, and bone marrow [1, 2]. It is the most common type of leukemia in adults, and symptoms include swollen lymph nodes, fatigue, infection, and night sweats [3, 4]. Though the disease can remain stable, it can also transform into aggressive lymphomas such as diffuse large B‐cell lymphoma (DLBCL) or Hodgkin lymphoma [1].

At the genetic level, several alterations have been identified in patients with B‐CLL, including mutations in *TP53, NOTCH1*, and the 13q14 deletion, which affects candidate tumor suppressor genes such as *DLEU2* and *DLEU7* [5, 6]. These abnormalities contribute to defining clinical and molecular subtypes that influence progression and response to treatment [7].

In parallel, epigenetics have emerged as a key determinant in the biology of B‐CLL. Epigenetic changes are those that influence gene expression without modifying the actual DNA sequence [2, 8]. Though important for normal development, epigenetic changes can cause uncontrolled growth in cancer [9]. In B‐CLL, these changes may occur through DNA methylation, histone modifications, and changes to noncoding RNA [2, 8, 10, 11]. For example, hypermethylation of tumor suppressor gene promoters can silence their expression, favoring tumor progression, whereas microRNA (miRNA) deregulation can alter key signaling pathways, such as PI3K/AKT or Notch [12, 13]. Because almost 20% of B‐CLL patients do not harbor chromosomal abnormalities, it is extremely important to look at the epigenetics involved in the disease [14].

B‐CLL is divided into three separate subtypes based on genetic and epigenetic signatures: memory‐like CLL (m‐CLL), intermediate CLL (i‐CLL), and naïve‐like CLL (n‐CLL). The m‐CLL is associated with mutated immunoglobulin heavy‐chain variable region (*IGHV)* (M‐CLL), whereas unmutated *IGHV* (U‐CLL) more closely resembles n‐CLL [1, 15]. The i‐CLL, a subtype intermediate to U‐CLL and M‐CLL, exhibits features of both subtypes and has intermediate *IGHV* mutational levels as well as heterogeneous epigenetic patterns [15].

Possible treatments for B‐CLL include demethylation agents and histone deacetylase inhibitors (HDACi) [8]. HDACi in particular have been shown to improve prognosis when used in conjunction with Ibrutinib, a BTK inhibitor approved by both the US Food and Drug Administration (FDA) and the European Medicines Agency (EMA) [1, 16].

Some epigenetic alterations have been observed to confer resistance to treatments such as BTK inhibitors or alkylating agents, thus understanding the epigenetic mechanisms involved in the evolution of B‐CLL is essential for establishing new therapeutic strategies, improving risk stratification, and moving toward personalized medicine [17, 18].

### 1.1. *IGHV* and *IGLV3-21^R110^*

**Figure 1:**
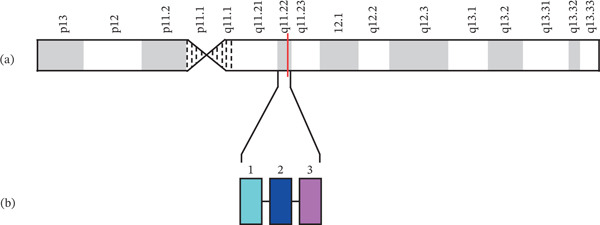
(a) Locus of *IGLV3-21* gene on 22q11.22 and (b) diagram of *IGLV3-21* gene on 22q11.22. Modified from [19, 20].

**Figure 2:**
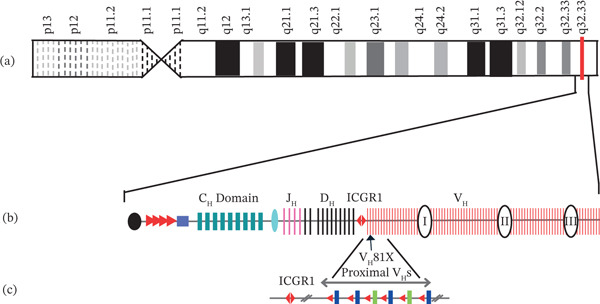
(a) Locus of *IGHV* gene on 14q32.33, (b) diagram of the IGH locus showing the V_H_, D_H_, J_H_, and C_H_ exons and regulatory elements (not to scale), and (c) the V_H_7183 and V_H_Q52 families, represented by blue and lime green bars, respectively, are located at the D_H_J_H_ proximal end of the locus. V_H_ gene segment names indicate their position along the locus. V_H_81X is the second gene segment relative to intergenic control Region 1 (IGCR1). Modified from [19, 21].

Two important genes associated with B‐CLL are immunoglobulin lambda variable 3‐21 *(IGLV3-21*) and *IGHV*. As shown in Figures 1 and 2, *IGLV3-21* is located at 22q11.22 and is believed to be involved in immune response, whereas *IGHV* is a group of 129 genes in the IGH locus located at 14q32.33 (Figure 1 [22–24]).

CLL is classified into three main subtypes: unmutated *IGHV* (U‐CLL), mutated *IGHV* (M‐CLL), and borderline *IGHV* (BL‐CLL) [15, 25]. U‐CLL, with ≥ 98% identity with the germline, originates from B cells that have not gone through the GC, whereas M‐CLL comes from B cells that have [1, 2]. These two subtypes have distinct biological features and clinical behaviors, with the former generally associated with a shorter time to first treatment (TTFT) and poorer prognosis and the latter with a longer TTFT and better prognosis [26]. In addition, about 30% of CLL patients show stereotyped B‐cell receptor (BCR) rearrangements, in which some extremely similar amino acid sequences in immunoglobulin gene rearrangements have been associated with specific clinical behaviors and more aggressive disease, especially in Subtype 2 [1].

The existence of the third subtype, BL‐CLL, was recently discovered and thus only has been written about in four papers. Due to the low prevalence of cases in CLL (4.3%–7.5%), clinical outcomes of BL‐CLL are poorly defined [25]. The earliest study, performed in 2016 by Davis et al., found that BL‐CLL has 97.00%–98.99% identity with the germline as well as intermediate TTFT and overall survival (OS) [27]. However, in 2020, Raponi et al. defined BL‐CLL as 97.00%–97.99% identity with the germline and a similar TTFT to M‐CLL patients [28].

**Figure 3:**
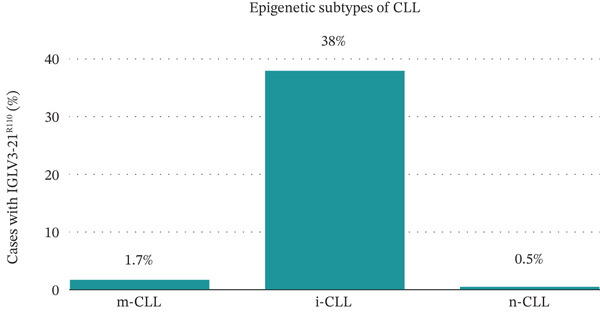
Percentages of cases in m‐CLL, i‐CLL, and n‐CLL with *IGLV3-21^R110^*. Modified from [15].

There are three epigenetic CLL subtypes based on their methylation signatures: m‐CLL, i‐CLL, and n‐CLL [2]. They are linked with *IGHV* mutation status, with m‐CLL having predominantly mutated *IGHV* and a favorable prognosis, whereas n‐CLL has predominantly unmutated *IGHV* and a poor prognosis [15]. The intermediate subtype, i‐CLL, contains cases with both mutated and unmutated *IGHV* and tends to have an intermediate prognosis. In addition, i‐CLL is distinguished by a higher usage of the *IGLV3-21* gene and often contains a mutation at position 110 (IGLV3‐21^R110^), which is associated with more aggressive disease and a poor prognosis. The *IGLV3-21^R110^* mutation, as a consequence of somatic hypermutation, occurs in 7%–18% of CLL and 38% of i‐CLL (Figure 3) [15]. This mutation has been linked to TTFT and OS, similar to that observed in n‐CLL [15]. This is because the *IGLV3-21^R110^* mutation causes autonomous BCR signaling and can upregulate *WNT5A*, a gene that is implicated in increased proliferation and chemotaxis of CLL cells [1].

The presence *of IGLV3-21^R110^* influences the behavior and prognosis of CLL irrespective of whether the *IGHV* gene is mutated or not. The *IGLV3-21^R110^* mutation was found in all cases with stereotype Subset 2, but although 62% of *IGLV3-21^R110^* cases lack stereotyped BCR immunoglobulins, the cases share the same genetic and transcriptomic characteristics [15]. On the other hand, i‐CLL cases that do not have the *IGLV3-21^R110^* mutation resemble m‐CLL cases, which are prognostically favorable, suggesting that the mutation plays a pivotal role in shaping the aggressive nature of i‐CLL [15]. The presence of the *IGLV3-21^R110^* mutation in BL‐CLL has not been tested in any previous work [25].

### 1.2. Epigenetic Mechanisms In B‐CLL and Their Relationship With Prognosis

#### 1.2.1. DNA Methylation and Regulation of Tumor Suppressor Genes

DNA methylation is one of the main epigenetic mechanisms regulating gene expression. This process involves the addition of methyl groups to cytosines within CpG sequences, which generally leads to transcriptional repression. Under physiological conditions, methylation is essential for processes such as embryonic development, X‐chromosome inactivation, and the suppression of transposable elements. However, in neoplasias such as B‐CLL, this methylation can become aberrant, silencing genes essential for cell cycle control and apoptosis, thus promoting the unregulated proliferation of malignant cells [18].

Hypermethylation of tumor suppressor gene promoters in the 13q14 region, such as *DLEU7*, has been observed in patients with B‐CLL [6]. This epigenetic alteration prevents the expression of these genes, which facilitates the clonal expansion of abnormal B lymphocytes and reduces their capacity to undergo apoptosis. The 13q14 region is particularly important in this disease, as it also houses the microRNAs miR‐15a/16‐1, whose deletion or silencing is closely associated with increased cell proliferation and apoptotic resistance [29].

Additionally, genes such as *CLLD6* and *RB1* have also been reported as frequent targets of aberrant methylation in B‐CLL. Inactivation of these genes not only promotes tumor cell survival but also contributes to genomic instability. Furthermore, there is evidence linking hypermethylation in this region with reduced efficacy in treatments such as chemotherapy or immunotherapy, which directly affects patient prognosis [18].

On the other hand, allelic loss (haploinsufficiency) has also been implicated in gene dysfunction in this region. The combination of partial deletions and aberrant methylation can generate a synergistic effect that exacerbates tumor progression [29].

Faced with this situation, therapeutic strategies aimed at reversing epigenetic changes have emerged. DNA methyltransferase inhibitors (DNMTi), such as azacitidine and decitabine, have shown efficacy in reactivating silenced suppressor genes. These agents act by incorporating themselves into DNA and blocking DNMT function, thereby allowing the reexpression of key genes involved in cell cycle regulation and apoptosis [11].

#### 1.2.2. Histone Modification and Chromosome Regulation

Posttranslational modifications of histones represent another essential epigenetic mechanism in gene regulation. Among these, acetylation, methylation, and phosphorylation are the most relevant, as they modulate chromatin structure and, consequently, DNA accessibility to transcription factors.

In B‐CLL, histone deacetylation, mediated by histone deacetylase enzymes (HDACs), has been associated with a poor prognosis. Loss of acetylation in histones H3 and H4 promotes chromatin compaction, generating a repressive environment for genes involved in apoptosis and cell cycle regulation [5]. This mechanism has been identified in genes located in the 13q14 region, such as *DLEU7*, *CLLD6*, and *RB1*, reinforcing their transcriptional silencing [6, 29].

Furthermore, studies have described mutations in chromatin regulatory genes such as ligand‐dependent nuclear receptor‐interacting factor 1 (*LRIF1*), whose normal function is linked to gene compaction and repression. Alterations in *LRIF1* could contribute to the progression of B‐CLL, even in the absence of changes in DNA methylation, highlighting the relevance of this epigenetic mechanism as an independent modulator [13].

From a therapeutic perspective, HDACi such as vorinostat and romidepsin have demonstrated clinical potential. These compounds promote histone reacetylation, reversing the repressive state of chromatin and reactivating the expression of tumor suppressor genes. Their use as epigenetic agents could be especially beneficial in patients with extensive epigenetic alterations [5].

#### 1.2.3. Noncoding RNA

MiRNAs, long noncoding RNAs (lncRNAs), and circular RNAs (circRNAs) play an important role in the epigenetics of CLL. MiRNAs are small noncoding RNA molecules that regulate gene expression at the posttranscriptional level by silencing or degrading messenger RNAs, thereby affecting essential processes such as cell proliferation, differentiation, and apoptosis [8, 30, 31]. In B‐CLL, miRNA dysregulation has emerged as a key factor in disease pathogenesis, progression, and prognosis.

**Figure 4:**
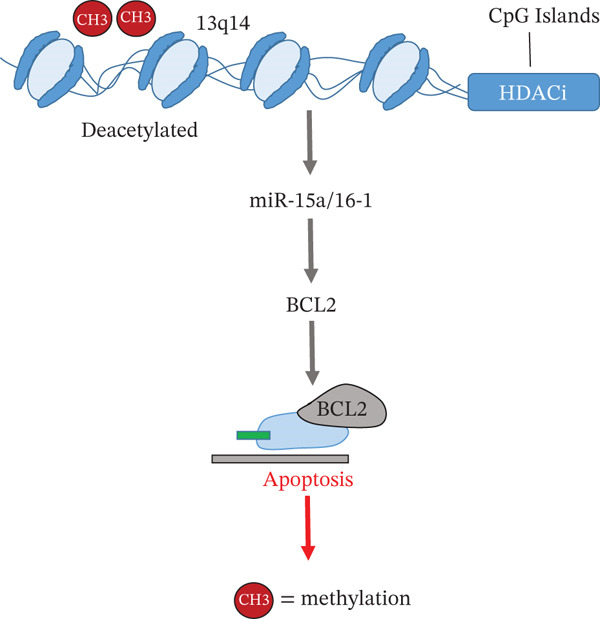
Epigenetic regulation of the miR‐15a/16‐1 cluster in the 13q14 region and its impact on BCL2 expression.

Among the most studied alterations in B‐CLL is the deletion of the 13q14 region, which includes miRNAs with tumor suppressor function, particularly miR‐15a and miR‐16‐1. This deletion, present in over 65% of cases, results in increased expression of BCL2 and other oncogenes, contributing to apoptotic evasion and enhanced survival of leukemic cells [6, 8, 29, 32]. As illustrated in Figure 4, this epigenetic silencing involves CpG island hypermethylation and histone deacetylation, which repress the expression of the miR‐15a/16‐1 cluster and promote BCL2 overexpression.

Hypermethylation of CpG islands and histone deacetylation induce transcriptional silencing of the miR‐15a/16‐1 cluster. This leads to BCL2 overexpression and reduced apoptosis in leukemic B cells.

Other miRNAs, such as miR‐29 and miR‐181, are also frequently deregulated in B‐CLL. Their decreased levels have been linked to sustained activation of oncogenic pathways, such as TCL1 and MCL1, which promote clonal expansion of abnormal B cells [11]. In contrast, miR‐155, whose overexpression has been associated with more aggressive forms of the disease, exerts its oncopromoting effect by inhibiting the expression of genes such as *SHIP1* and *C/EBPβ*, which are essential in the regulation of apoptosis and differentiation [5].

From a clinical perspective, miRNA expression profiling in B‐CLL has emerged as a promising prognostic and predictive biomarker. For instance, patients with deregulated expression of key tumor‐suppressive or oncomiRs may exhibit altered responses to chemotherapy and immunotherapy [29].

In this context, miRNA‐targeted therapies represent an innovative strategy. Efforts have focused on restoring the function of tumor suppressor miRNAs through mimics, as well as silencing oncogenic miRNAs such as miR‐155 using antagomirs. These approaches are being evaluated in preclinical and clinical trials, showing potential to complement conventional treatments in patients with B‐CLL (Table 1) [11].

**Table 1:** Functional characterization of microRNAs involved in B‐cell chronic lymphocytic leukemia (B‐CLL): loci, therapeutic targets, and clinical applications. Modified from [8].

MicroRNA	Locus	Expression and target gene	Biological function	Impact on prognosis of B‐CLL	Reference
MiR‐15/16	13q14	Down, *BCL2, MCL1*	Inhibits expression of *BCL2* and regulation of *TP53* to induce cell death	Unfavorable	[33, 34]
MiR‐29b‐3p	1q32.2	Down, *TCL-1, MCL-1*	Targets *TRAF4* to modulate CD40 signaling	Unfavorable	[35, 36]
MiR‐181a‐5p / miR‐181b‐5p	1q32.1/1q32.1	Down, *TCL-1, BCL-2*	Regulation of apoptotic pathways	Unfavorable	[37]
MiR‐34a/b/c	1p36.22	Down, *TP53, CDK4, CDK6*	Inhibits cell proliferation associated with the expression of *TP53* and *ZAP-70*	Unfavorable	[33, 38]
MiR‐17‐5p (miR‐17‐92)	13q31.3	Up, *PTEN, Bim*	Activation of cell proliferation	Unfavorable	[39]
MiR‐125a‐5p	11q24.1	Up/down, *MYC, BAK1, LIN28*	Predicts Richter syndrome development, microenvironment/immunity regulator	Favorable	[38]
MiR‐150‐5p	19q13.33	Down in worse prognosis, MYB, FOXP1	Modulates heterogeneity via regulating expression of *GAB1* and *FOXP1*	Favorable	[40]
MiR‐155‐3p	21q21.3	Up, *SHIP1, BCR*	Enhances BCR signaling	Unfavorable	[41, 42]
MiR‐222‐3p	Xp11.3	Down, multiple regulators	Potential tumor suppressor	Favorable	[12]
MiR‐146b‐5p	10q24.32	Up, NF‐*κ*B regulators	Antiproliferative/anti‐inflammatory	Unfavorable	[43]
MiR‐26a‐5p	3p22.2	Up, *CD38* (targeted delivery)	Induces selective apoptosis	Favorable	[44]
MiR‐193b/miR‐193a‐5p	16p13.12/17q11.2	Down, *CDK6, MCL1*	Cell cycle control	Unfavorable	[45]
MiR‐21‐5p	17q23.2	Up, *PTEN, PDCD4*	Classic OncomiR	Unfavorable	[46]
MiR‐92a‐3p	13q31.3	Up, differentiation genes	Cellular suppressor/regulator	Unfavorable	[17]
MiR‐148a‐3p	7p15.2	Down, *BCL2, DNMT1*	Proapoptotic inhibitor	Unfavorable	[47]
MiR‐223‐3p	Xq12	Up, *STMN1, FOXO3A*	Differentiation control	No impact	[12]
MiR‐146a‐5p	5q33.3	Up, *EGR1, TRAF6*	Anti‐inflammatory/regulation of differentiation	Favorable	[37]
MiR‐142‐5p	17q22	Up, *BAFFR, GFI1*	Regulates development and function B	Unfavorable	[48]
MiR‐412	14q32.31	Down, unknown	Not clearly defined	Unfavorable	[49]
MiR‐324‐3p	17p13.1	Up, unknown	Not clearly specified	Favorable	[50]

##### 1.2.3.1. MiR‐15a/16‐1.

The miR‐15a/16‐1 cluster is located at 13q14, one of the most commonly affected regions in B‐CLL. The deletion of 13q14 is the most frequent genetic abnormality in these patients [51, 52]. This region, also referred to as the minimal deleted region (MDR), includes the noncoding gene DLEU2 and the miRNAs miR‐15a‐5p and miR‐16‐1‐5p, whose loss or dysfunction has significant implications in the initiation, progression, and remodeling of the immunological microenvironment of the disease [51, 52].

At the molecular level, miR‐15a/16‐1 functions as a key tumor suppressor by negatively regulating genes involved in cell proliferation and survival. Its best documented target is *BCL2*, a central regulator of apoptosis [51, 52]. Loss of this regulatory axis enhances resistance to cell death in neoplastic B cells, promoting their clonal expansion and persistence in protective niches. Furthermore, their role extends beyond apoptosis regulation to other essential aspects of tumor biology.

In murine models, targeted deletion of miR‐15a/16‐1 disrupts germinal center (GC) homeostasis, increasing the population of GC B cells and favoring their differentiation into plasma cells. Over time, this dysregulation results in the development of mature B‐cell neoplasms such as extramedullary plasmacytoma, follicular lymphoma, and DLBCL [52]. These experimental findings are mirrored in humans, where primary extramedullary plasmacytoma patients exhibit low miR‐15a/16 expression and a high frequency of 13q deletions, supporting its context‐dependent tumor suppressor role [52].

Likewise, miR‐15a/16‐1 plays a crucial role during the early stages of B‐cell development, controlling progenitor proliferation by inhibiting cell cycle–related genes such as *Ccne1*, *Ccnd3*, *Cdc25a*, and the interleukin‐7 receptor (*IL7R*) [53]. The simultaneous loss of the miR‐15a/16‐1 and miR‐15b/16‐2 clusters leads to an abnormal expansion of pro‐B and pre‐B cell populations through activation of the IL7R/PI3K/AKT pathway, creating a pro‐proliferative and potentially preleukemic environment [53].

Additionally, this cluster regulates the immune microenvironment. In models with deletion of the MDR, the loss of miR‐15a/16‐1 promotes the differentiation of monocytes into M2‐type macrophages with immunosuppressive properties, and it is associated with PD‐L1 overexpression in both immune and leukemic cells [51]. These alterations generate an immunologically dysfunctional microenvironment, favoring tumor immune evasion and disease progression.

Clinically, the loss of miR‐15a/16‐1 has been associated with a more indolent subtype of B‐CLL, particularly in patients with isolated del(13q). However, its functional expression may be reduced even in the absence of deletion, limiting its utility as a standalone prognostic biomarker [12, 51]. From a therapeutic standpoint, miR‐15a/16‐1 mimetics encapsulated in nanoparticles have been developed to restore its tumor‐suppressive function. Furthermore, its reactivation has been suggested to enhance sensitivity to venetoclax, a selective *BCL2* inhibitor approved for B‐CLL [51].

The biogenesis of miR‐15a/16‐1 may follow either a canonical or noncanonical pathway. In the canonical route, it is transcribed as a pri‐miRNA and processed by the Drosha‐DGCR8 complex; in the noncanonical pathway, it arises as a mirtron processed by the spliceosome. The pre‐miRNA is subsequently exported to the cytoplasm by Exportin‐5 and cleaved by Dicer together with TRBP and Argonaute proteins, forming the RISC complex. This complex allows the mature miRNA to interact with *BCL2* for translational repression or degradation [10, 11].

Altogether, the miR‐15a/16‐1 cluster plays a multifaceted role in B‐CLL, being involved not only in the direct regulation of apoptosis but also in early lymphocytic development and the shaping of the tumor microenvironment. Its loss is not only a frequent structural alteration but also represents a functional axis whose restoration may offer significant therapeutic benefits.

**Figure 5:**
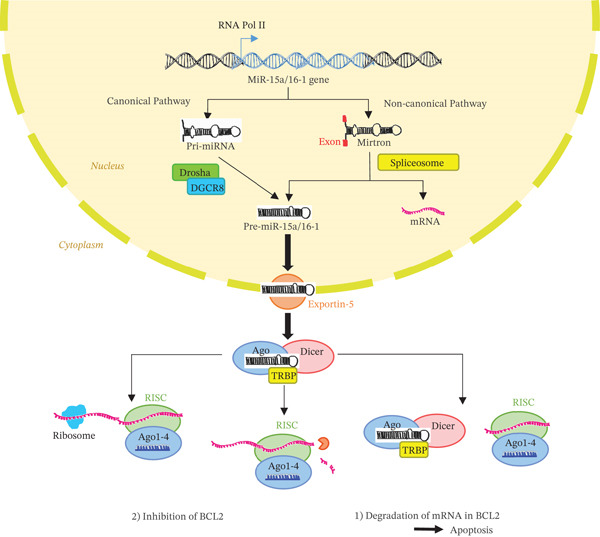
Biogenesis of the miR‐15a/16‐1 cluster and its action on BCL2 in B cells. Modified from [54].

Figure 5 illustrates both the canonical and noncanonical pathways involved in the biogenesis of the miR‐15a/16‐1 cluster. In the canonical pathway, RNA Polymerase II transcribes the pri‐miRNA, which is processed by the Drosha–DGCR8 complex into pre‐miR‐15a/16‐1. This is then exported to the cytoplasm by Exportin‐5 and cleaved by Dicer–TRBP to form a mature miRNA duplex. In the noncanonical pathway, mirtrons are generated via splicing and directly enter the Dicer processing step. The mature miRNA is incorporated into the RNA‐induced silencing complex (RISC), which uses Argonaute proteins (Ago1–4) to mediate posttranscriptional gene silencing [54].

miR‐15a/16‐1 targets the 3 ^′^UTR of *BCL2* mRNA, leading to either mRNA degradation (Pathway 1) or translational inhibition (Pathway 2), resulting in decreased BCL2 protein levels and the induction of apoptosis. This regulatory mechanism is essential in maintaining normal B‐cell homeostasis and is frequently disrupted in B‐CLL [54].

##### 1.2.3.2. MiR‐29.

Another central miRNA in the epigenetic regulation and tumor progression of B‐CLL is miR‐29b‐3p, a member of the miR‐29 family, which also includes miR‐29a and miR‐29c. This family is highly evolutionarily conserved, and its loss of expression has been associated with more aggressive forms of the disease, underscoring its role as a tumor suppressor [11, 12, 17, 51]. Reduced levels of miR‐29b‐3p are linked to a rapidly progressing clinical phenotype, even in patients without high‐risk mutations such as *TP53*.

At the molecular level, miR‐29b‐3p negatively regulates multiple oncogenes overexpressed in leukemic B cells, including *TCL1*, *MCL1*, and *TRAF4*, which promote proliferation, inhibit apoptosis, and contribute to resistance to conventional therapies [11, 17]. It also modulates key signaling pathways such as PI3K/AKT and nuclear factor (NF)‐*κ*B, directly affecting tumor‐associated inflammation and the maintenance of the neoplastic phenotype [12, 55, 56]. Its influence also extends to the leukemic microenvironment, where it has shown antiproliferative and antiangiogenic effects, possibly through endothelial cells associated with the tumor niche.

Clinically, low miR‐29b‐3p expression has been associated with higher tumor burden, therapeutic resistance, and shorter progression‐free survival (PFS) [11, 17, 51]. Conversely, its overexpression in murine models has led to less aggressive phenotypes and reduced clonal expansion, suggesting a potential role in risk stratification, especially in patients with rapidly evolving disease but without evident mutational biomarkers.

From a therapeutic perspective, the restoration of miR‐29b‐3p in cell models has shown synergy with BCR receptor inhibitors such as ibrutinib, increasing sensitivity to apoptosis and reducing the expression of prosurvival genes [11]. This finding has driven the development of miR‐29b‐3p mimetics as a potential adjuvant therapeutic strategy, particularly in patients with suboptimal responses to targeted therapies.

In summary, miR‐29b‐3p represents a multifunctional regulatory axis in B‐CLL, impacting both intrinsic tumor biology and the leukemic microenvironment. Its profile positions it as a prognostic biomarker and a promising target for next‐generation epigenetic interventions, reinforcing its inclusion in personalized strategies for disease monitoring and treatment.

##### 1.2.3.3. MiR‐181.

Continuing with miRNAs that play key roles as tumor regulators in B‐CLL, miR‐181a‐5p and miR‐181b‐5p stand out. Both are derived from the miR‐181a/b‐1 cluster, located on the long arm of chromosome 1 (1q31.3). Because they share the same seed sequence, they have common molecular targets, allowing them to coordinately modulate multiple critical pathways involved in apoptosis, proliferation, and immune signaling [57]. In this context, they negatively regulate genes such as *BCL2*, *TCL1*, and various components of the NF‐*κ*B pathway, reinforcing their role as tumor suppressors in B‐CLL [37, 58].

The expression of miR‐181a/b‐5p has been shown to decrease progressively with the clinical advancement of the disease, supporting their use as dynamic biomarkers of tumor progression. Clinical studies have revealed that low levels of miR‐181b‐5p are associated with higher tumor burden, early treatment requirement, resistance to anti‐CD20 therapies, and increased relapse rates [11, 17, 37]. Serial monitoring of their expression in longitudinal samples has proven useful for distinguishing indolent from aggressive forms of the disease, aiding in individualized risk stratification [37].

At the experimental level, overexpression of miR‐181a/b‐5p has shown antiproliferative and proapoptotic effects in cellular models, accompanied by the inhibition of genes such as *LMO3*, *PTEN*, *SNAI2*, and *WIF1*, which are involved in aberrant differentiation and tumor progression processes [58]. Animal models have confirmed that the reintroduction of miR‐181a‐5p through mimetics not only reverses chemoresistance phenotypes but also reduces pathological angiogenesis, primarily via modulation of the PI3K/AKT and TGF‐*β* pathways [59, 60].

Altogether, miR‐181a‐5p and miR‐181b‐5p stand out as multifunctional elements with diagnostic, prognostic, and therapeutic applications. Their broad regulatory impact on key leukemic physiopathological pathways positions them as strong candidates for noninvasive monitoring strategies, mimetic‐based or antagomiR‐based therapies, and integration into personalized medicine platforms for B‐CLL.

##### 1.2.3.4. MiR‐34.

Among the group of miRNAs regulated by TP53, miR‐34a‐5p occupies a central position due to its role as a key effector of the tumor suppressor pathway. Encoded on chromosome 1p36, this member of the miR‐34 family (which also includes miR‐34b and miR‐34c) regulates multiple genes involved in cell cycle control and apoptosis such as *CDK4*, *CDK6*, *BCL2*, *FLOT2*, and *NOTCH2*, promoting G1‐phase arrest and inducing programmed cell death [61–63].

In B‐CLL, miR‐34a‐5p expression is markedly decreased in patients with 17p deletion or TP53 mutations, which impairs its transcription and allows uncontrolled proliferation of neoplastic cells [61, 64]. However, reduced levels have also been documented in individuals without detectable TP53 mutations but with clinical resistance to fludarabine, suggesting a functional or epigenetic disruption of the TP53–miR‐34a axis that escapes standard genetic diagnostic methods [64].

From a clinical standpoint, low levels of miR‐34a‐5p are associated with poor prognosis, reduced response to chemotherapy, and an increased risk of clonal transformation into aggressive variants such as Richter′s syndrome [62, 64]. Serial evaluation of its expression could enable early detection of relapse and provide complementary prognostic information to conventional cytogenetics, particularly in patients with genomic instability.

Functional studies have shown that overexpression of miR‐34a‐5p in murine models and B‐CLL cell lines inhibits proliferation, induces G1 arrest, and reduces the expression of *CDK6* and *CCND1*. Its direct binding to the 3 ^′^UTR region of *CDK6* has been experimentally validated as a key mechanism of its tumor‐suppressive action [63]. In parallel, highly stable synthetic mimetics have demonstrated antileukemic effects even in models with dysfunctional TP53, opening new therapeutic possibilities for high‐risk patients [63, 64].

In summary, miR‐34a‐5p functions as a central mediator connecting epigenetics, the cell cycle, and the TP53 pathway. Its functional loss represents a critical alteration in B‐CLL biology. Its potential as a high‐risk biomarker and as a therapeutic target for mimetic‐based strategies positions it as a key tool in personalized medicine, particularly in scenarios of therapeutic resistance or clonal transformation.

##### 1.2.3.5. MiR‐17‐92.

The miR‐17‐92 cluster, located on the long arm of Chromosome 13 (13q31.3), comprises six mature miRNAs—miR‐17‐5p, miR‐18a, miR‐19a, miR‐20a, miR‐19b‐1, and miR‐92a‐1—and represents one of the most extensively characterized oncogenic clusters in both hematologic and solid malignancies [39]. In B‐CLL, this cluster is highly expressed in the most aggressive clinical forms, with miR‐17‐5p being the most relevant component due to its direct effects on genes regulating apoptosis and cell proliferation [65].

At the molecular level, miR‐17‐5p represses key tumor suppressor genes such as *PTEN*, *BIM (BCL2L11)*, and *CCNG2*, thereby promoting cell survival and evasion of apoptosis [66, 67]. This repression enables accelerated cell cycle progression into the G2/M phase, particularly through the negative modulation of E2F‐dependent inhibitors, which enhances the clonal expansion of neoplastic B lymphocytes [39, 67]. Overexpression of the full cluster in hematological models also leads to constitutive activation of signaling pathways such as PI3K/AKT and MYC, promoting a more aggressive leukemic phenotype [65].

Clinically, miR‐17‐5p overexpression correlates with high‐risk features such as CD38 and ZAP70 positivity, as well as unfavorable cytogenetic abnormalities, higher tumor burden, and resistance to therapies including fludarabine or BCR inhibitors [65]. These associations support its role as an independent prognostic marker and a potential predictor of therapeutic response.

Recently, an additional function of miR‐17‐5p has been described as a transcriptional activator through the RNA activation (RNAa) mechanism, promoting the expression of *KPNA2* and repressing proapoptotic genes such as *BAX*, *Caspase-3*, and *Caspase-9* [68]. This finding suggests a dual role in gene regulation that extends beyond the classical model of posttranscriptional inhibition, with possible implications for tumor adaptation to the leukemic microenvironment.

Given its role in disease progression and therapy resistance, preclinical strategies are being explored to selectively inhibit miR‐17‐5p using antagomiRs or other specific molecules, aimed at restoring the balance between proliferation and apoptosis in patients with refractory disease.

Altogether, miR‐17‐5p and the miR‐17–92 cluster constitute a key functional unit within the epigenetic architecture of B‐CLL. Its sustained overexpression profile, impact on critical regulatory genes, and involvement in emerging gene activation mechanisms position it as a high‐value therapeutic target, particularly in settings of rapid disease progression or resistance to conventional therapies.

##### 1.2.3.6. MiR‐155.

MiR‐155‐3p is the lesser‐studied strand of the miR‐155 precursor, encoded by the *BIC* gene (MIR155HG) located at chromosome 21q21.3. Although long considered a degraded strand, recent evidence has revealed that miR‐155‐3p possesses specific functional activity, particularly in immunological contexts and in the regulation of the tumor microenvironment [69, 70].

In B‐CLL, miR‐155‐3p plays a pro‐oncogenic role, primarily by enhancing BCR signaling through the direct inhibition of *SHIP1 (INPP5D)*, a key negative regulator of the PI3K/AKT pathway [69]. This sustained activation of the BCR pathway promotes proliferation, apoptosis evasion, and maintenance of the malignant phenotype. Additionally, miR‐155‐3p represses tumor suppressor genes such as *SOCS1*, *FBXW7*, and *TP53INP1*, reinforcing its oncogenic profile [69, 70].

From a clinical perspective, miR‐155‐3p overexpression has been associated with aggressive forms of B‐CLL, with elevated levels correlating with resistance to BTK inhibitors such as ibrutinib, shorter PFS, and higher relapse rates [69]. Recently, its plasma detection has been proposed as a noninvasive liquid biomarker for dynamic disease monitoring and assessment of therapeutic response [71, 72]. Technological tools such as electrochemical microfluidic sensors with molecular beacons have enabled ultrasensitive and specific detection, showing promising potential for clinical application [72].

In the immunological setting, miR‐155‐3p regulates multiple components of the tumor microenvironment, including dendritic cell activation, macrophage polarization, and suppression of effective T‐cell responses [70]. Its expression is induced by toll‐like receptors (TLRs), Type I interferons, and chronic inflammatory stimuli, positioning it as a molecular link between sustained inflammation and malignant transformation. It has also demonstrated effects on endothelial cells and regulatory T cells, facilitating processes such as angiogenesis and persistent immune activation.

At the experimental level, specific inhibitors and antagomiRs targeting miR‐155‐3p are currently under development to restore therapeutic sensitivity and counteract pathological signaling [69]. However, because it shares a precursor with miR‐155‐5p, as well as its potential for dynamic arm‐switching, more refined characterization is required to minimize off‐target effects.

Altogether, miR‐155‐3p emerges as a multifunctional regulator with direct impact on tumor signaling, immune modulation, and clinical progression in B‐CLL. Its elevated expression represents a robust marker of poor prognosis, and its specific inhibition may offer an alternative therapeutic route for patients with refractory disease or relapse after immunotherapy.

##### 1.2.3.7. MiR‐222‐3p.

MiR‐222‐3p is a mature strand derived from the miR‐221/222 cluster, encoded on chromosome Xp11.3, a region involved in key cellular processes including proliferation, apoptosis, and therapy resistance [12]. Although most literature has focused on its role in solid tumors, recent evidence has begun to uncover its functional relevance in hematologic malignancies, including B‐CLL.

At the molecular level, miR‐222‐3p functions as an apoptosis modulator by directly inhibiting *PUMA (BBC3)*, a proapoptotic effector of the mitochondrial pathway, thereby allowing cells to evade programmed cell death and enhance survival [73]. This activity contributes to persistent clonal expansion and accumulation of therapy‐resistant cells. Additionally, miR‐222‐3p represses other genes such as *ZEB1*, affecting cellular plasticity and enabling adaptive mechanisms in response to immunologic or therapeutic signals [68]. Although these effects have primarily been described in solid tumor models, the underlying mechanisms are extrapolatable to the microenvironmental context of B‐CLL, given the shared role of these pathways in tumor regulation.

Although direct data in B‐CLL remain limited, experimental studies have shown that miR‐222‐3p may be downregulated in certain hematologic contexts, and that its suppression promotes apoptosis and reduces cell proliferation [12]. These findings suggest a potential dual behavior depending on the cellular and immunological context, particularly in chronically activated B cells, and may be influenced by factors such as the tumor microenvironment, mutational burden, and pharmacological pressure.

From a clinical perspective, miR‐222‐3p has been proposed as an emerging poor prognostic biomarker, with potential application in risk stratification and therapeutic monitoring. Its detection in biological fluids such as plasma has been explored as a noninvasive tool for tracking tumor progression, particularly in chronic diseases with an inflammatory component [74]. However, more robust validation in specific B‐CLL cohorts is still needed to consolidate its clinical utility.

Altogether, miR‐222‐3p represents a functionally relevant miRNA that regulates key genes involved in apoptotic evasion and tumor plasticity. Although its role in B‐CLL is still exploratory, its molecular profile suggests potential prognostic value and promise as a therapeutic target in selected disease scenarios.

##### 1.2.3.8. MiR‐146b‐5p.

On the other hand, miR‐146b‐5p, encoded on chromosome 10q24.32 by the *MIR146B* gene, belongs to the same family as miR‐146a, with which it shares the seed sequence, although it displays a distinct expression pattern and functional behavior [75]. In B‐CLL, it has been identified as an epigenetic modulator with relevant immunoregulatory roles within the tumor microenvironment.

One of its most notable mechanisms involves the posttranscriptional inhibition of the *IL23R* and *IL12RB1* subunits, which are required for the formation of the functional interleukin‐23 receptor (IL‐23R), thereby interfering with downstream cytokine signaling [43]. This pathway has been linked to the expansion of pro‐inflammatory leukemic clones, making its modulation a critical point in disease progression. In B‐CLL cellular models, overexpression of miR‐146b‐5p has resulted in decreased IL‐23–induced proliferation and a significant reduction in the activation of STAT3, a key mediator of tumor promotion [43].

Clinically, although miR‐146b‐5p has not yet been established as an independent prognostic marker, reduced levels may be associated with a more active tumor microenvironment, particularly in patients with overexpression of the IL‐23R complex. These findings have spurred interest in exploring therapeutic strategies aimed at restoring its expression, with the goal of limiting the proliferation of leukemic subclones stimulated by pro‐inflammatory cytokines.

In summary, miR‐146b‐5p is a miRNA with key functions in the regulation of IL‐23–mediated and STAT3‐mediated signaling, positioning it as a potential biomarker of tumor‐associated inflammation and a strategic therapeutic target. Its incorporation into molecular monitoring platforms could be particularly useful in personalized approaches aimed at intervening at the interface between inflammation and leukemic progression.

##### 1.2.3.9. MiR‐26a‐5p.

Along the same lines, miR‐26a‐5p is another miRNA with notable roles in regulating proliferation and the tumor microenvironment. Encoded by the loci MIR26A1 (3p22.2) and MIR26A2 (12q14.1), this miRNA is ubiquitously expressed and participates in key cellular processes, including cell cycle control, inflammatory signaling, and the immune response [44].

At the molecular level, miR‐26a‐5p predominantly exerts tumor‐suppressive effects by inhibiting genes such as *PTEN*, *EZH2*, *CCND2*, *PRXIII*, and *WNT5A*, interfering with pro‐oncogenic pathways including PI3K/AKT, WNT/*β*‐catenin, and epigenetic mechanisms [44, 76–79]. Although these effects have been extensively documented in solid tumors, their implications in B‐CLL have recently begun to be explored with encouraging results.

One relevant application in B‐CLL has been the development of therapeutic strategies based on the selective delivery of miR‐26a‐5p to CD38+ cells, a subpopulation associated with poor prognosis. This approach has demonstrated the ability to induce tumor apoptosis without affecting normal cells, positioning it as a promising alternative in cases refractory to fludarabine or BCR inhibitors such as ibrutinib [44].

From a clinical standpoint, elevated levels of miR‐26a‐5p have been correlated with better prognosis and increased treatment sensitivity in hematologic models, whereas its loss may be associated with greater tumor aggressiveness and a dysregulated inflammatory microenvironment [62, 77, 78]. Its detection and quantification could provide additional prognostic value in risk stratification frameworks.

At the experimental level, the use of miR‐26a‐5p mimetics has shown the ability to reverse malignant phenotypes and reduce inflammatory mediators such as *CTGF*, *IL-6*, and *TNF-α*, suggesting an immunomodulatory function that complements its role in proliferation control [62]. These properties strengthen its potential as a dual therapeutic target—both antitumoral and immunoregulatory.

Altogether, miR‐26a‐5p emerges as a clinically relevant miRNA in B‐CLL, with a multifunctional profile that makes it a strong candidate for targeted therapies, especially in patients with high CD38 expression or prior therapy resistance. Its validation in clinical studies could open new avenues in personalized medicine for chronic leukemias.

##### 1.2.3.10. MiR‐125b‐5p.

Continuing with dual‐function miRNAs in B‐CLL, miR‐125b‐5p stands out for its ambivalent role in tumor and immune regulation. This mature strand is derived from two independent loci—*MIR125B1* (11q24.1) and *MIR125B2* (21q21.1)—and shows highly conserved expression in hematopoietic cells [80]. Its activity regulates key processes such as inflammation, proliferation, and immune evasion, making it a critical node in tumor homeostasis.

In the specific context of B‐CLL, miR‐125b‐5p has been described as a functionally versatile miRNA, capable of acting as either a tumor suppressor or promoter depending on the cell type, microenvironment, and mutational burden [80]. One of its best characterized mechanisms is the direct inhibition of *TRAF6*, an essential modulator of NF‐*κ*B and MAPK pathway activation induced by *IL-1β*, leading to the repression of proinflammatory genes such as *COX2*, *IL-6*, and *TNF-α* [81]. This action suggests an immunoregulatory effect that may help counteract the inflammatory microenvironment typical of B‐CLL.

In addition, miR‐125b‐5p influences adaptive immunity by inhibiting *TNFR2* in regulatory T cells (Tregs), thereby reducing their immunosuppressive activity and enhancing CD8+ T‐cell activation, ultimately promoting a more effective immune response against tumor cells [82]. This property positions it as a key modulator in advanced stages of the disease, which are often characterized by pronounced immune evasion.

From a clinical perspective, miR‐125b‐5p overexpression has been associated with both more active immune profiles and reduced tumor‐associated inflammation, depending on the cellular context [80, 82]. These findings suggest its potential as a biomarker for immune stratification or immunotherapy response, although broader clinical validation is still needed to confirm its prognostic value.

Therapeutically, strategies have been proposed based on mimetics or delivery vectors aimed at transporting miR‐125b‐5p directly into the tumor microenvironment, with the goal of restoring immune surveillance in combination with immunotherapies or anti‐inflammatory agents. This approach may be particularly useful in refractory B‐CLL, where immune dysfunction limits the efficacy of conventional treatments.

##### 1.2.3.11. MiR‐193b/miR‐193a‐5p.

Among the group of tumor‐suppressive miRNAs, miR‐193a‐5p and miR‐193b belong to the same functional family and share high sequence conservation, despite being encoded at different loci: MIR193A (17q11.2) and MIR193B (16p13.12) [45]. Both exert epigenetic control over key processes such as proliferation, migration, and cell survival, and their dysregulation has been observed in both solid tumors and hematologic malignancies.

At the molecular level, miR‐193a‐5p functions as an inhibitor of proleukemic signaling pathways by directly suppressing *SPOCK1*, a gene associated with PI3K/AKT and MAPK/ERK activation—pathways linked to clonal survival, tumor progression, and therapy resistance [45, 83]. This inhibition not only slows down cell proliferation but also reduces invasive potential, offering a relevant therapeutic advantage.

miR‐193b, in turn, enhances therapeutic sensitivity by activating proapoptotic axes such as TP53 and BAX/BAK, while also modulating genes associated with drug resistance and tumor metabolism [45]. This sensitizing effect has been particularly valued in refractory leukemia, where restoring cell death pathways is a critical strategy to counteract tumor evasion.

From a clinical standpoint, decreased expression of both miR‐193a‐5p and miR‐193b has been documented in B‐CLL patients, correlating with poorer prognosis, higher aggressiveness, and lower response to conventional therapies [45, 83]. This dysregulation has led to their inclusion in proposed molecular risk panels, particularly due to their association with imbalances in inflammatory and metabolic pathways.

Recently, miR‐193a‐5p has been considered a candidate in clinical trials due to its impact on key intracellular signaling pathways and its regulatory effects on the leukemic microenvironment [84]. Its potential as a biomarker of therapeutic response and as an immunotherapy adjuvant remains under evaluation, but it represents a promising avenue for personalized epigenetic intervention.

Altogether, miR‐193a‐5p and miR‐193b stand out as epigenetic regulators with antiproliferative, anti‐inflammatory, and proapoptotic activity. Their functional profile makes them valuable tools for risk stratification, therapeutic monitoring, and the design of targeted strategies in B‐CLL.

##### 1.2.3.12. MiR‐21‐5p.

Within the spectrum of oncogenic miRNAs, miR‐21‐5p is among the most extensively studied for its broad involvement in both hematologic and solid neoplasms. Its gene is located at 17q23.2, within the intron of the *TMEM49* gene, suggesting a co‐expression mechanism regulated by growth and differentiation signals [46]. In B‐CLL, its overexpression has been linked to key processes such as cell proliferation, apoptosis inhibition, tumor microenvironment remodeling, and treatment resistance.

The mechanism of action of miR‐21‐5p is based on the posttranscriptional repression of tumor suppressor genes such as *PTEN*, *SPRY2*, *PDCD4*, *KRIT1*, and *TGFBI*. By inhibiting *PTEN*, the PI3K/AKT pathway is disinhibited, enhancing cell survival. Simultaneously, *KRIT1* suppression promotes *β*‐catenin activation, stimulating tumor angiogenesis and vascular permeability—two processes that facilitate clonal dissemination and B‐CLL progression [85]. This influence over multiple molecular pathways consolidates its role as a central epigenetic regulator in this neoplasm.

Additionally, miR‐21‐5p has been found to be enriched in tumor‐derived exosomes, from which it modulates the leukemic microenvironment by altering immune responses and promoting a permissive vascular context. This exosomal signaling contributes to both immune evasion and the establishment of therapeutic resistance, highlighting its role as a key mediator of intercellular communication in B‐CLL [46, 85].

Clinically, elevated levels of miR‐21‐5p in serum and exosomes have been associated with greater tumor aggressiveness, chemoresistance, and poor prognosis. Its expression correlates with reduced OS and increased risk of relapse, which has led to its proposal as a noninvasive biomarker for diagnosis, progression monitoring, and evaluation of therapeutic response [46, 85].

In preclinical studies, the specific inhibition of miR‐21‐5p using antagomiRs has reversed tumor phenotypes and restored sensitivity to cytotoxic agents, opening the possibility of incorporating it as a therapeutic target. Its functional, clinical, and experimental profile reinforces its importance among epigenetically active miRNAs in B‐CLL and suggests that its blockade could serve as a complementary tool in the design of personalized therapies.

##### 1.2.3.13. MiR‐92a‐3p.

miR‐92a‐3p, a member of the miR‐17~92 cluster, is located at 13q31.3, within Intron 3 of the *MIR17HG* gene. This cluster has been extensively characterized as a key oncogenic regulator in multiple malignancies, including B‐CLL, where its activation is associated with more aggressive phenotypes [17].

From a functional perspective, miR‐92a‐3p promotes cell proliferation and resistance to apoptosis by inhibiting tumor suppressor genes such as *PTEN*. This suppression disinhibits the PI3K/AKT/mTOR pathway, supporting clonal survival and tumor progression [86]. In addition, miR‐92a‐3p has been implicated in regulating the cisplatin response, where it activates antiapoptotic mechanisms that increase treatment resistance. Although this function has been better documented in other cancer types, similarities in signaling pathways allow for reasonable extrapolations to the B‐CLL context [87].

Clinically, miR‐92a‐3p expression has been detected in plasma samples, and it has been proposed as a noninvasive biomarker with potential utility for the diagnosis and monitoring of cancer patients. Its circulating overexpression has been associated with worse prognosis and greater aggressiveness, reinforcing its potential predictive value [88]. In B‐CLL, although direct studies remain limited, the miR‐17~92 cluster has been shown to be overexpressed in patients with high CD38 and *ZAP70* expression, suggesting the active involvement of miR‐92a‐3p in the more unfavorable subtypes of the disease [17].

Altogether, miR‐92a‐3p is a functional component of a cluster with significant epigenetic impact in B‐CLL. Its action on *PTEN* and the activation of key pro‐oncogenic pathways position it as a relevant candidate for targeted therapeutic interventions, particularly in cases with poor prognosis or refractory to standard treatments.

##### 1.2.3.14. MiR‐148a‐3p.

MiR‐148a‐3p, located at 7q22.1, has been identified as a relevant epigenetic regulator, with roles in modulating immune responses, DNA repair, and chemoresistance. Although its specific profile in B‐CLL has not been fully characterized, the molecular mechanisms described in other tumor types suggest its possible involvement in this neoplasm.

Functionally, miR‐148a‐3p acts as a tumor suppressor by inhibiting the expression of genes such as *IGF-IR*, *IRS1*, *RAD51*, *HMGB1*, and *PD-L1*—all involved in critical pathways of proliferation, DNA repair, immune evasion, and therapeutic response [47, 89–91]. In particular, the repression of *RAD51*, a key protein in homologous recombination, may increase cellular sensitivity to chemotherapeutic agents such as cisplatin, suggesting a mechanism of resensitization in treatment‐resistant neoplasms [91].

A particularly noteworthy aspect is its immunomodulatory capacity. The inhibition of *PD-L1* by miR‐148a‐3p may help restore antitumor immune surveillance, a process often suppressed in B‐CLL to enable disease progression [47, 92]. Additionally, silencing of *HMGB1*, an inflammatory protein associated with therapy resistance and tumor remodeling, suggests a therapeutic benefit by targeting the immunosuppressive microenvironment and improving sensitivity to radiotherapy and chemotherapy [90].

In summary, miR‐148a‐3p emerges as a clinically relevant miRNA in B‐CLL due to its involvement in key processes such as immune evasion, DNA repair, and chemoresistance. Although more disease‐specific experimental validation is needed, its functional profile supports its consideration as an emerging prognostic biomarker and a promising therapeutic target, with potential application in combined or personalized therapies.

##### 1.2.3.15. MiR‐150‐5p.

On the other hand, miR‐150‐5p, located on 19q13, is predominantly expressed in mature hematopoietic cells and plays an essential role in lymphoid differentiation, especially of B lymphocytes. Its dysregulation has been linked to various neoplasms, and in B‐CLL, a significant reduction in its expression has been documented, associated with a more aggressive clinical course and poor prognosis.

At the molecular level, miR‐150‐5p acts as a tumor suppressor by directly inhibiting the oncogene *MYB*, a central regulator of cell proliferation and survival. The downregulation of miR‐150‐5p leads to *MYB* overexpression, promoting clonal expansion, increasing therapeutic resistance, and contributing to a more advanced tumor phenotype [93]. This epigenetic alteration results in the loss of physiological control over differentiation and apoptotic mechanisms.

Clinically, miR‐150‐5p has been proposed as both a diagnostic and prognostic biomarker, not only due to its differential expression in tumor tissue but also because of its detection in peripheral blood, allowing for noninvasive assessments to monitor disease progression or identify early‐stage disease [94]. Its expression profile has shown correlation with clinical outcomes and tumor burden, reinforcing its value as a risk stratification tool.

From a therapeutic perspective, the restoration of miR‐150‐5p using synthetic mimetics or viral vectors has been explored with the aim of reducing *MYB* activity and restoring immune control mechanisms in the leukemic microenvironment. This approach has been shown to enhance immunotherapy efficacy and improve the antitumor response, suggesting complementary value in combined treatment regimens [95, 96].

Altogether, miR‐150‐5p represents a key epigenetic regulator whose loss promotes tumor progression in B‐CLL. Its functional and clinical profile supports its consideration as a prognostic biomarker and emerging therapeutic target, especially in refractory settings or those with poor response to conventional treatments.

##### 1.2.3.16. MiR‐223‐3p.

Along the same lines, miR‐223‐3p, located on Chromosome X (Xp11.3), is highly expressed in myeloid cells such as neutrophils and monocytes. It plays an active role in the regulation of hematopoiesis, inflammatory response, and innate immunity, acting through posttranscriptional silencing by binding to the 3 ^′^UTR regions of target mRNAs, thereby preventing their translation or promoting degradation [12].

In the context of B‐CLL, miR‐223‐3p exerts a tumor‐suppressive effect by inhibiting genes such as *FBXW7* and *MEF2C*, both of which are involved in the regulation of cell proliferation and activation of inflammatory pathways like NF‐*κ*B [49]. Its overexpression has been shown to reduce cell viability and limit the invasive capacity of leukemic cells, whereas its downregulation promotes more aggressive and treatment‐resistant phenotypes [48].

Clinically, elevated levels of miR‐223‐3p in serum or tumor tissue have been associated with more favorable prognosis, lower tumor burden, and better therapeutic response. This differential expression supports its use as a prognostic biomarker and as a complementary criterion for risk stratification in B‐CLL patients. Moreover, its functional profile positions it as a potential tool for guiding more personalized therapeutic interventions.

Altogether, miR‐223‐3p represents a miRNA with immunoregulatory and antiproliferative functions, whose profile in B‐CLL makes it an attractive candidate for the development of more precise diagnostic and therapeutic strategies, aligned with the principles of personalized medicine.

##### 1.2.3.17. MiR‐146a‐5p.

In the same category of immunomodulatory and tumor‐suppressive miRNAs, miR‐146a‐5p is located on chromosome 5q33.3, a region broadly associated with the regulation of immune and inflammatory processes. This miRNA acts as a key regulator of the innate immune response, modulating cytokine signaling and TLR pathways [97].

Its mechanism of action in B‐CLL focuses on the inhibition of the NF‐*κ*B pathway through posttranscriptional silencing of regulatory genes such as *TRAF6* and *IRAK1*. This suppression reduces chronic inflammatory signaling that promotes cell proliferation, apoptosis evasion, and tumor progression. Although in other neoplasms it has also been associated with inhibition of epithelial–mesenchymal transition (EMT), in B‐CLL, its predominant function relates to the regulation of inflammatory and survival pathways [97].

From a clinical standpoint, elevated levels of miR‐146a‐5p have been correlated with increased sensitivity to agents such as fludarabine, an effect attributed to its modulation of the PTEN/PI3K/AKT pathway, which is central to antineoplastic therapy resistance. These findings support its utility as both a prognostic and predictive biomarker, as well as its potential as a therapeutic target within personalized medicine strategies [98].

##### 1.2.3.18. MiR‐142‐5p.

Continuing with miRNAs involved in the regulation of the immune microenvironment and tumor progression, miR‐142‐5p is located on chromosome 17q22, within the 17q22–q23 region. Its expression is predominantly hematopoietic, playing a critical role in the development, differentiation, and function of immune cells such as B and T lymphocytes and dendritic cells [48].

In the context of chronic lymphocytic leukemia of B cells (B‐CLL), miR‐142‐5p acts as a posttranscriptional regulator of genes involved in immune signaling, cell survival, and tumor microenvironment remodeling. Its main target genes include *TGFBR1*, a component of the TGF‐*β* pathway, and *ZEB2*, a repressor of *E-cadherin*. Dysregulation of miR‐142‐5p promotes persistence of the malignant lymphoid phenotype by facilitating both immune escape and cellular migration [49, 50].

Clinically, increased expression of miR‐142‐5p has been observed in advanced stages of B‐CLL compared with normal B lymphocytes. This overexpression has been linked to poor prognosis, greater biological aggressiveness, and resistance to treatments such as fludarabine [51]. Its detection in peripheral blood and bone marrow has been proposed as a tool for risk stratification and therapeutic monitoring.

Moreover, functional studies have shown that overexpression of miR‐142‐5p enhances cell viability and reduces apoptosis, promoting clonal expansion of leukemic cells. Thus, inhibition of miR‐142‐5p emerges as a promising therapeutic strategy aimed at restoring sensitivity to cytotoxic drugs and reducing tumor burden [48].

##### 1.2.3.19. MiR‐412.

Focusing on miRNAs whose deregulation contributes to loss of proliferative control in B‐CLL, miR‐412 is located on chromosome 14q32, specifically within the miR‐379/miR‐656 cluster, a locus involved in epigenetic regulation and the development of various malignancies, including B‐CLL [52].

Regarding its mechanism of action, miR‐412 regulates genes associated with cell proliferation and apoptosis. In B‐CLL, its plasmatic expression is decreased, possibly reflecting a loss of regulation over tumor‐suppressor pathways. This deregulation indirectly affects key genes related to cell cycle and immune response, favoring the clonal expansion of malignant B cells [53].

Clinically, low miR‐412 expression at diagnosis has been associated with poorer clinical outcomes and lower treatment response. Therefore, its assessment—together with other key miRNAs—has been proposed as a valuable tool for prognostic stratification in B‐CLL patients from the beginning of therapeutic intervention [52].

##### 1.2.3.20. MiR‐324‐3p.

In the same context, miR‐324‐3p is located on chromosome 17p13.1, a region known to host genes involved in cell cycle control and apoptosis. This miRNA has been identified as a tumor suppressor in several types of cancer through negative regulation of genes associated with cell proliferation and survival [54, 55].

Regarding its mechanism of action, miR‐324‐3p primarily functions through posttranscriptional silencing of oncogenic genes. For example, studies in cervical and pancreatic cancer have shown that this miRNA inhibits cell proliferation by interfering with the miR‐324‐3p/ELK1 axis and the expression of LINC01320, a lncRNA that acts as a competing endogenous RNA (ceRNA), blocking its natural regulatory function [54, 55]. Likewise, recent research has shown that miR‐324‐3p is part of disrupted ceRNA networks in B‐CLL, contributing to tumor progression in this context [56].

As for its prognostic impact, the expression profile of miR‐324‐3p has been proposed as a component of risk prediction models in B‐CLL patients, as part of epigenetic gene signatures that include other miRNAs, lncRNAs, and coding genes. These molecular stratification models could improve personalized therapeutic decision‐making, highlighting its value as an emerging biomarker in this neoplasm [56]. Although therapy resistance directly linked to miR‐324‐3p has not yet been reported, its role in regulating proliferation and apoptosis pathways suggests it may be included in future epigenetic modulation–based therapeutic strategies.

#### 1.2.4. LncRNA in B‐CLL

LncRNAs have over 200 nucleotides, and several studies have highlighted their role in regulating pathogenic mechanisms in cancer [8, 99]. Two crucial lncRNAs are DLEU1 and DLEU2 (Table 2). Though further research is needed to establish their exact roles in CLL, they are believed to help produce tumor suppressors that block NF‐*κ*B activity. DLEU1 is also notable as the host gene for miR‐15/16 [8, 30].

**Table 2:** Functional characterization of long noncoding RNAs involved in B‐cell chronic lymphocytic leukemia (B‐CLL): loci, therapeutic targets, and clinical applications. Modified from [8].

LncRNA	Locus	Expression and target gene	Biological function	Impact on prognosis of B‐CLL	Reference
AC092652.2–202	2q22.2‐q22.3	Up, *TP53*	Related to ultra conserved Region 70 and has prognostic significance	Unfavorable	[100]
BM742401	18q11.2	Down, *GATA6*	Inhibits cell proliferation and induces cell death	Unfavorable	[30, 101]
CRNDE	16q12.2	Down, *IRX5*	Inhibits cell proliferation via miR‐28/*NDRG2* axis and regulated by DNA methylation	Unfavorable	[102]
DLEU1/2	13q14	Down, *RB1, BCL2*	Involved in the inhibition of NF‐*κ*B activity	Favorable if restricted to MDR, unfavorable if part of large deletion	[30]
Lnc‐IRF2‐3	4q35.1	Up in *IGHV* unmutated, *ZAP70*	Associated with primary immunodeficiency	Unfavorable	[103]
Lnc‐KIAA1755‐4	20q11.23	Up	Involved in ribosome formation and translational processes	Unfavorable	[103]
Lnc‐LEF1‐AS1	4q25	Up, *LEF1*	Interacts with *LEF1* and promotes cell proliferation	Unfavorable	[104]
LincRNA‐p21	6p21.2	Typically down but up in *TP53* wild‐type	Induces cell death after DNA damage	Downregulation is associated with an unfavorable prognosis	[105]
Lnc‐TOMM7‐1	7p15.3	Down‐regulated in early stage, *IL6*	Antisense to *IL6* and promotes cell death	Possible unfavorable	[103]
NEAT1	11q13.1	Up in *TP53* wild‐type	Involved in transcriptive process activated by p53 and DNA damage	Correlates with better prognostic markers but is not an independent predictor of favorable outcome	[106]

##### 1.2.4.1. AC092652.2–202.

AC092652.2‐202 is overexpressed in B‐CLL patients and is associated with a shorter TTFT and unfavorable prognosis [105]. It is important to note that its inferior clinical outcome is independent of *IGHV* mutational status. AC092652.2‐202 may influence genes involved in key cancer pathways such as NF‐*κ*B activation, *TP53*, and apoptosis [100, 105]. In addition, its expression is also linked to how patients respond to CpG‐ODN treatment, further suggesting that this lncRNA plays a functional role in disease behavior and treatment response [100].

##### 1.2.4.2. BM742401.

BM742401 is located on chromosome 18q11.2 and is positioned antisense to the *GATA6* gene [14, 105]. It functions as a tumor suppressor, but it is often silenced in B‐CLL through methylation of its promoter region, leading to an unfavorable prognosis [14, 100]. On the other hand, normal B cells show no methylation in this region, indicating that the methylation is specific to B‐CLL and may contribute to the disease.

BM742401 functions via the intrinsic, *Caspase-9*–dependent pathway, rather than the extrinsic, *Caspase-8* pathway. Its methylation is found in about 40% of primary B‐CLL samples at diagnosis, and it is associated with reduced expression of *GATA6*. Low levels of *GATA6* are thought to contribute to disease progression [10]. Additionally, BM742401 methylation in CLL patients correlates with more advanced disease features, including higher lymphocyte counts and older age at diagnosis. It is also frequently observed alongside methylation of miR‐129‐2 [105].

Treatment with hypomethylating agent 5‐Aza‐2 ^′^‐deoxycytidine (decitabine) has been shown to increase BM742401 expression and promote apoptosis [14].

##### 1.2.4.3. CRNDE.

Also known as LINC00180, CRNDEP, or lincIRX5, CRNDE is located on the opposite strand of chromosome 16q12.2. It is positioned close to the *IRX5* gene, which is involved in cell cycle control and apoptosis, and both are thought to share a bidirectional promoter, which may coordinate their expression [14, 105, 107]. CRNDE is significantly downregulated in CLL cell lines such as MEC‐1 and HG3, as well as in primary CLL samples when compared with normal B lymphocytes [102].

Treatment with 5‐Aza‐2 ^′^‐deoxycytidine reduced methylation levels and reactivated CRNDE expression, leading to a noticeable reduction in leukemia cell proliferation and increased apoptosis in both MEC‐1 and HG3 cell lines. CRNDE exerts its antileukemic effects primarily through the miR‐28/*NDRG2* signaling axis [108]. miR‐28 normally suppresses *NDRG2*, a tumor suppressor gene involved in cell growth and apoptosis. By inhibiting miR‐28, CRNDE indirectly allows *NDRG2* levels to rise, which slows down cell proliferation and promotes programmed cell death. This suggests that CRNDE plays a tumor‐suppressive role in CLL, and its downregulation contributes to disease progression [102].

Low CRNDE expression in CLL patients was found to correlate with the presence of B symptoms, low hemoglobin and platelet levels, high total leukocyte and lymphocyte counts, elevated serum LDH and *β*2‐microglobulin levels, and the presence of del17p, a known poor‐prognosis genetic marker. Kaplan–Meier survival analysis also confirmed that patients with hypermethylated CRNDE had significantly shorter OS [109].

##### 1.2.4.4. DLEU1/2.

DLEU1 and DLEU2, along with the miR‐15a/16‐1 miRNA cluster, are found in the 13q14 chromosomal region. They are transcribed in opposite directions, and their first exons lie within the MDR of 13q14, making them highly susceptible to deletion in CLL [105].

The 13q14 deletion has different prognostic implications depending on its size. When the deletion is small and restricted to the MDR, affecting only DLEU1, DLEU2, and miR‐15a/16‐1, it is actually associated with a favorable prognosis in CLL. Patients with this subtype of deletion tend to have longer TTFT and better OS [14]. However, larger deletions that extend beyond the MDR to include other genes, especially *RB1* (retinoblastoma gene), are linked to a worse prognosis. These patients typically experience shorter PFS and OS due to the loss of additional tumor suppressor functions [14].

DLEU2 is the host gene for miR‐15a and miR‐16‐1, and when it is deleted or downregulated, miR‐15a/16‐1 levels drop, leading to increased expression of cyclins and BCL2 [8]. This causes enhanced proliferation and resistance to apoptosis [105].

DLEU1 and DLEU2 may also exert independent regulatory functions. Recent studies suggest that these lncRNAs influence the expression of neighboring protein‐coding tumor suppressor genes, which are also located at 13q14. These genes are involved in modulating the NF‐*κ*B signaling pathway, which controls inflammation, cell survival, and immune responses [14, 105].

Furthermore, epigenetic studies have shown that the transcriptional start sites of DLEU1 and DLEU2 are hypomethylated in CLL cells compared with normal B cells, a change often associated with increased transcriptional activity [105]. However, this hypomethylation correlates positively with the expression of DLEU1 and DLEU2 but negatively with the expression of nearby tumor suppressors, indicating a tightly regulated, perhaps competing, expression system. This in cis coregulation suggests that DLEU1 and DLEU2 may also act by modulating the chromatin environment to influence gene activity in their neighborhood [105].

##### 1.2.4.5. Lnc‐TOMM7‐1, Lnc‐IRF2‐3, and Lnc‐KIAA1755‐4.

Lnc‐TOMM7‐1 is located on Chromosome 7p, antisense to the *IL6* gene [103]. More studies are needed to establish its prognostic impact, but current research suggests it is downregulated in CLL and may be associated with a worse prognosis. *IL6* acts as an autocrine growth factor in CLL, promoting the proliferation and differentiation of B cells. Because lnc‐TOMM7‐1 is thought to regulate *IL6* transcription, its reduced expression may contribute to disease progression [103, 105].

Lnc‐IRF2‐3, located on chromosome 4q35, is consistently overexpressed in high‐risk CLL patients, particularly those with unmutated IGHV, high *ZAP70* expression, high Binet and Rai stages, and the del17p chromosomal abnormality [110]. These features are all known adverse prognostic markers. Furthermore, patients with elevated lnc‐IRF2‐3 levels had significantly shorter OS and PFS. Kaplan–Meier analyses confirmed that lnc‐IRF2‐3 is a strong negative prognostic biomarker in CLL [105, 110].

Lnc‐IRF2‐3 shows the highest expression in cases with NOTCH1 mutations (which is associated with aggressive disease), and it may also induce apoptosis via regulation of the BAX/Bcl‐2 ratio [110].

Lnc‐KIAA1755‐4, derived from an intron of the snoRNA host gene 17 (located on Chromosome 20q11.23), is found in the nucleolus and is involved in regulating rRNA modifications [105]. Its upregulation is associated with an unfavorable prognosis. One study analyzed patients with low expression of lnc‐KIAA1755‐4 and lnc‐IRF2‐3 and discovered that they correlated with a long TTFT and better prognosis [105].

##### 1.2.4.6. Lnc‐LEF1‐AS1.

Analysis of lncRNA expression profiles revealed that LEF1‐AS1 is significantly upregulated in primary CLL cells compared with normal B cells [104, 105]. This upregulation was further confirmed in a cohort of newly diagnosed CLL patients, where LEF1‐AS1 levels were consistently higher than in healthy controls. However, no clear association was found between LEF1‐AS1 expression and established clinical or prognostic markers at diagnosis [105].

Functional studies using CLL cell lines demonstrated that overexpression of LEF1‐AS1 leads to increased cell survival and reduced apoptosis, suggesting it may promote leukemia progression [105]. Moreover, LEF1‐AS1 appears to enhance the expression of LEF1, a gene known to be involved in CLL and associated with poor prognosis when highly expressed [104]. The connection between LEF1‐AS1 and LEF1 was reinforced by experiments showing that LEF1‐AS1 not only upregulates LEF1 at both RNA and protein levels but also physically binds to the LEF1 protein [104, 105].

These findings suggest that LEF1‐AS1 may exert its oncogenic effects by directly regulating and interacting with LEF1, enhancing signaling pathways that favor leukemic cell survival [105]. However, more studies are needed to confirm its prognostic significance.

##### 1.2.4.7. LincRNA‐p21.

LincRNA‐p21 is transcribed in the opposite direction from the *CDKN1A* gene and produces a 3.1 kb transcript with two exons [105]. When DNA damage occurs in various tumor cell lines, the lincRNA‐p21 promoter is activated by p53, resulting in its transcription. In CLL cells with a normal *TP53* gene, lincRNA‐p21 expression is strongly upregulated. However, in cells with 11q deletions or *TP53* deletions or mutations, lincRNA‐p21 expression is downregulated, which is associated with a poor prognosis [14, 111].

LincRNA‐p21 influences gene expression by binding to hnRNP‐K, which then targets the promoters of genes that are corepressed by p53 and lincRNA‐p21. If lincRNA‐p21 is disrupted, p53 activity is repressed, and apoptosis may be blocked [14].

LincRNA‐p21 also interacts directly with MDM2, promoting the MDM2‐p53 complex and forming a feedback loop that regulates p53′s activity [111]. Both lincRNA‐p21 and p53 are involved in regulating genes responsible for apoptosis [105]. When both lincRNA‐p21 and p53 are reduced, proapoptotic genes are suppressed whereas antiapoptotic genes are activated, suggesting that lincRNA‐p21 is part of the process that leads to cell death after DNA damage. However, lincRNA‐p21 does not significantly affect the transcription of *CDKN1A* or other cell cycle regulators, and it does not contribute to cell cycle arrest, unlike p53 [30, 105].

##### 1.2.4.8. NEAT1.

NEAT1 is transcribed into two isoforms: NEAT1_1, a short, polyadenylated transcript (~3.7 kb), and NEAT1_2, a longer, nonpolyadenylated transcript (~23 kb) that includes the entire NEAT1_1 sequence. NEAT1_2, in particular, is essential for building paraspeckles and has been linked to cell stress response, DNA repair, apoptosis, and chemoresistance [106].

The *TP53* gene, which encodes the tumor suppressor protein p53, is often altered in CLL—about 10% of newly diagnosed CLL patients carry a deletion in Chromosome 17p where *TP53* is located. This frequency increases as the disease progresses [30].

In response to DNA damage, wild‐type p53 (TP53wt) activates several lncRNAs, including NEAT1 and LincRNA‐p21 [30]. Although the direct involvement of these lncRNAs in CLL is not fully understood, they are known to be part of the *TP53*‐mediated DNA damage response [14]. NEAT1 induction leads to paraspeckle formation, which helps limit DNA damage and modulate p53 activity, ultimately influencing whether the cell survives or undergoes apoptosis [30, 112].

Studies have shown that NEAT1 expression increases in CLL cells exposed to DNA‐damaging agents, but only if *TP53* is functional [106, 112]. In CLL cells with *TP53* deletions or mutations, NEAT1 induction is impaired. Additionally, NEAT1 levels correlate with *p21*, another p53 target gene, and with cell viability. Cells unable to upregulate NEAT1 upon DNA damage tend to die more readily, pointing to a protective role for NEAT1 in stressed but repair‐capable cells [14, 105].

One study analyzed NEAT1 expression in a group of newly diagnosed Binet A CLL patients, and the researchers discovered that overall NEAT1 expression in CLL was not significantly different from normal B cells [105]. However, NEAT1_2 expression was higher in cases with *IGHV* mutations, 13q deletion, or no cytogenetic abnormalities and lower in cases with trisomy 12 [105, 106].

Patients with the lowest NEAT1_2 levels had the shortest TTFT, but this was not independent of other established prognostic markers like IGHV mutational status [105]. Still, NEAT1, particularly NEAT1_2, remains an important lncRNA to be studied in CLL.

#### 1.2.5. CircRNA in B‐CLL

Lastly, circRNAs feature closed ends formed through back‐splicing [30]. Though their expression is frequently changed in cancer, they were not known to be involved in CLL until a 2016 study by Macchia et al. showed upregulation of cMYC due to PTV1 and circPTV1 [113]. Since then, the most important circRNAs in CLL have been identified as circ‐CBFB, circ‐RPL15, and circ‐0132266 (Table 3) [30].

**Table 3:** Functional characterization of circular RNAs involved in B‐cell chronic lymphocytic leukemia (B‐CLL): loci, therapeutic targets, and clinical applications. Modified from [8].

CircRNA	Locus	Expression and target gene	Biological function	Impact on prognosis of B‐CLL	Reference
Circ‐0002078	Not linked to a specific gene	Up, *TCF7L1*	Regulates *TCF7L1* expression to promote cell proliferation	Unfavorable	[108]
Circ‐0132266	6q13	Down	Promotes cell viability through miR‐337‐3p/PML axis	Unfavorable	[114]
Circ‐CBFB	16q22.1	Up, *FZD3*	Increases cell proliferation as well as prognostic and diagnostic marker	Unfavorable	[115]
Circ‐RPL15	3p24.2	Up	Increases cell proliferation and diagnostic biomarker	Unfavorable	[116]
CircZNF91	19p12	Up, *WEE1*	Promotes malignant phenotype by targeting miR‐1283/*WEE1* axis	Unfavorable	[98]
Mc‐COX2 (Mt‐circRNAs)	Found on plasma exosome	Up	Promotes cell proliferation and inhibits apoptosis	Unfavorable	[117]

##### 1.2.5.1. Circ‐0002078.

Circ‐0002078 regulates *TCF7L1* expression by sponging miR‐185‐3p [108]. MiR‐185‐3p normally suppresses *TCF7L1*, a gene that promotes cell growth, so its inhibition leads to high levels of *TCF7L1* expression as well as B‐CLL cell growth and survival. A 2022 study by Zhang et al. discovered that high circ‐0002078 expression in CLL patients is linked to shorter OS and a worse prognosis [108]. Furthermore, gene ontology (GO) analyses in the study showed that circ‐0002078′s target genes are involved in lymphocyte proliferation and B cell survival, which are critical for CLL progression. Kyoto Encyclopedia of Genes and Genomes (KEGG) analyses also revealed that the target genes are involved in the JAK‐STAT signaling pathway and cytokine signaling [108].

##### 1.2.5.2. Circ‐0132266.

Circ‐0132266, found on chromosome 6q13, functions as a tumor suppressor in B‐CLL by promoting cell death through the miR‐337‐3p/ pro–myelocytic leukemia protein (PML) pathway [108]. By sponging miR‐337‐3p, circ‐0132266 indirectly helps maintain levels of PML, a protein that helps suppress tumors [30]. Circ‐0132266 is underexpressed in CLL, which leads to an unfavorable prognosis [114].

##### 1.2.5.3. Circ‐CBFB.

Overexpression of circ‐CBFB (16q22.1) is associated with a poor prognosis as it promotes cancer cell growth and blocks cell death [108]. Circ‐CBFB sponges miR‐607, a miRNA that blocks *FZD3*. *FZD3* is involved in the Wnt/*β*‐catenin signaling pathway—a pathway that helps cancer cells grow and survive [115]. Essentially, by blocking miR‐607, circ‐CBFB increases *FZD3* levels, which activates the Wnt/*β*‐catenin pathway and promotes CLL cell growth and survival. On the other hand, circ‐CBFB knockdown suppressed B‐CLL cell proliferation and caused cell death [115].

##### 1.2.5.4. Circ‐RPL15.

Circ‐RPL15 sponges miR‐146b‐3p, a miRNA that helps prevent cancer growth by blocking *RAF1.* Upregulation of *RAF1* activates the RAS/RAF1/MEK/ERK pathway [30, 116]. Because this pathway promotes tumor growth and survival, higher levels of circ‐RPL15 are linked to worse prognosis [116]. Additionally, circ‐RPL15 is positively associated with *IGHV* mutation status (circ‐RPL15 is particularly overexpressed in patients with unmutated *IGHV*) [108].

##### 1.2.5.5. CircZNF91.

CircZNF91 is found in the cytoplasm of MEC‐1 and HG‐3 [98]. When overexpressed in B‐CLL, it is associated with an unfavorable prognosis. When circZNF91 is silenced, it slows down the proliferation of CLL cells, induces apoptosis, and causes cell cycle arrest. CircZNF91 binds to and inhibits miR‐1283, which normally suppresses the expression of *WEE1*, a protein that regulates the cell cycle. By binding to miR‐1283, circZNF91 leads to increased *WEE1* expression, promoting the survival and continued growth of B‐CLL cells. However, the exact mechanism of how circZNF91 works is still unknown [98].

##### 1.2.5.6. Mc‐COX2.

Mc‐COX2 is a mitochondrial circRNA that is highly expressed in the plasma exosomes of B‐CLL patients. Its upregulation is associated with cell proliferation, inhibition of apoptosis, and decreased mitochondrial function [117].

### 1.3. Mutations in Transcription Factors and Epigenetic Regulators

Mutations in genes encoding transcription factors and epigenetic regulators play a critical role in the progression of B‐CLL. These alterations modify the expression of multiple critical cellular pathways, impacting the proliferation, differentiation, and survival of malignant cells. Among the most studied genes are *NOTCH1* and *LRIF1*, whose dysfunction has been associated with more aggressive phenotypes, decreased therapeutic response, and adverse outcomes [11].

The *NOTCH1* gene encodes a transmembrane receptor essential for the regulation of B cell homeostasis and differentiation. In patients with B‐CLL, *NOTCH1* mutations are identified in approximately 10%–12% of cases at diagnosis, and their frequency can reach up to 30% in those with transformation to Richter syndrome, an aggressive variant of the disease [5]. The most frequent mutations affect the PEST region, responsible for the regulated degradation of the intracellular portion of the receptor (NICD). Its loss leads to sustained activation of the NOTCH pathway, promoting the transcription of genes such as *CCND3*, *MYC*, and *BCL2*, all of which are involved in cell proliferation and resistance to apoptosis [5].

In addition to its role as a transcriptional activator, *NOTCH1* has also been shown to be involved in epigenetic regulation, inducing changes in chromatin structure through histone modulation. This dual capacity reinforces its involvement in the progression of B‐CLL and suggests a therapeutic avenue for intervention in patients with activating mutations.

On the other hand, *LRIF1* is an epigenetic regulator involved in chromatin compaction and gene silencing. Mutations in *LRIF1* have been identified in patients with B‐CLL, which have been associated with greater clonal stability and persistence of the leukemic population over time [11]. This stability may favor the accumulation of secondary mutations and contribute to therapeutic resistance. *LRIF1* acts by interacting with chromatin remodeling complexes, so its functional loss can trigger the derepression of genes that promote cell proliferation.

In clinical terms, the presence of *NOTCH1* mutations has been linked to a decreased response to immunological therapies, especially those using monoclonal antibodies such as rituximab. Furthermore, they confer a higher risk of progression to aggressive forms such as Richter syndrome [5]. Similarly, *LRIF1* dysfunction has been linked to a more resistant phenotype, possibly due to the activation of aberrant genetic programs that promote cell survival and hinder treatment response [11].

These observations have prompted research into NOTCH pathway inhibitors as a therapeutic alternative. These agents could block aberrant receptor activation and limit clonal proliferation, opening up new possibilities for personalized treatment of B‐CLL.

### 1.4. Therapeutic Implications and Strategies Based on Epigenetics

Given the significant impact of epigenetics on the progression of B‐CLL, therapeutic strategies have been developed to reverse these alterations. DNMTi, such as azacitidine and decitabine, have shown efficacy in reactivating tumor suppressor genes silenced by aberrant methylation, thereby restoring control mechanisms over the cell cycle and apoptosis [34]. Complementarily, HDACi, such as vorinostat and romidepsin, facilitate chromatin relaxation and reexpression of epigenetically repressed genes, including those located in the critical 13q14 region, which is frequently altered in B‐CLL [5].

Furthermore, posttranscriptional regulation by miRNAs has emerged as a relevant therapeutic target. The use of miR‐15a/16‐1 mimics or miR‐155 inhibitors represents a promising strategy to restore the altered epigenetic balance, enhance chemosensitivity, and induce apoptosis in leukemic cells [11]. Additionally, the NOTCH1 signaling pathway—often aberrantly activated due to mutations in its PEST domain—has also been explored as a therapeutic target. Specific inhibitors of this pathway are under development, aimed at blocking its sustained oncogenic effect and at improving therapeutic response, especially in patients with more aggressive disease phenotypes [5].

## 2. Conclusions

Epigenetic alterations are a key factor in the pathophysiology of B‐CLL, modulating cell survival, proliferation, and therapeutic resistance. Understanding them has helped explain some of the clinical heterogeneity observed in patients, while also opening up new therapeutic opportunities.

The integration of epigenetic biomarkers, such as methylation profiles, miRNA expression, or mutations in regulatory genes, could optimize risk stratification, enabling personalized medicine based on each patient′s molecular characteristics. In this context, epigenetic therapeutic strategies are emerging not only as a complement but also as a potential pillar in the treatment of B‐CLL, with a view to improving clinical outcomes and patients′ quality of life.

## Funding

No funding was received for this manuscript.

## Conflicts of Interest

The authors declare no conflict of interest.

## Data Availability

The data that supports the findings of this study are available in the supplementary material of this article.
